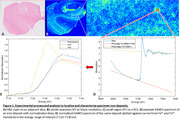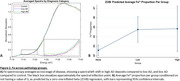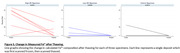# X Ray Absorption Near Edge Spectroscopy (XANES) Shows Abnormal Iron Oxidation State in Alzheimer’s Hippocampi

**DOI:** 10.1002/alz.088089

**Published:** 2025-01-03

**Authors:** Dean Tran, Phillip DiGiacomo, Jeffrey Nirschl, Nicholas Edwards, Sharon Bone, Samuel Webb, Marios Georgiadis, Michael Zeineh

**Affiliations:** ^1^ Stanford University, Stanford, CA USA; ^2^ SLAC Linear Accelerator National Laboratory, Menlo Park, CA USA

## Abstract

**Background:**

Recent studies suggest that iron and neuroinflammation are key components of Alzheimer’s Disease (AD) pathology. Ferrous Fe^2+^ can cause oxidative stress and cellular toxicity, but it is unknown to what extent Fe^2+^ is elevated in AD, in particular with the hippocampus. To answer this question, we quantified iron oxidation state in frozen human brain hippocampi.

**Methods:**

22 fresh‐frozen human brain hippocampi from the Stanford Alzheimer’s Disease Research Center (high AD—n = 13, low AD—n = 7, non‐AD controls—n = 2) were imaged in beamline 7‐2 of Stanford’s Synchrotron Radiation Lightsource using X‐ray fluorescence imaging (XFI) to identify regions of interest (ROIs) with high iron concentration (**Fig. 1A‐B**). X‐ray absorption near‐edge structure (XANES) spectroscopy was performed on ROIs to determine the relative proportions of Fe^2+^ and Fe^3+^ in each deposit (**Fig. 1B‐E**). A Fischer’s exact test was performed to evaluate the association between AD stage and levels of Fe^2+^. Since Fe^2+^ proportion is a bounded measure with an excess of zeroes, a zero/one‐inflated beta (ZOIB) regression model was fitted.

In addition, to interrogate the effects of tissue thawing on iron oxidation state during the hours long scan times, a cryo‐chamber kept 3 hippocampal specimens frozen while their respective deposits were being scanned. These specimens were then allowed to thaw and their same deposits rescanned. The proportion of Fe^2+^ between the paired frozen/thawed deposits were compared using a Wilcoxon signed‐rank test.

**Results:**

There was a statistically significant increase in Fe^2+^ in the AD specimens (p = 0.007). The ZOIB regression showed that low AD (z = 9.10, p<0.001) and high AD (z = 3.36, p = 0.001) hippocampi exhibit a significantly higher proportion of Fe^2+^ compared to controls.

Thawed deposits showed a statistically significant lower proportion of Fe^2+^ compared to their frozen state (p = 0.0145).

**Conclusions:**

We found increased Fe^2+^ in thawed AD hippocampi compared to controls. Additionally, the thawing of frozen iron deposits resulted in a decrease of measured Fe^2+^. These findings may suggest actual differences are even higher between iron oxidation state in AD hippocampi versus controls.